# Motivating students’ participation in a computer networks course by means of magic, drama and games

**DOI:** 10.1186/2193-1801-3-362

**Published:** 2014-07-16

**Authors:** Constantinos S Hilas, Anastasios Politis

**Affiliations:** Department of Informatics and Communications, Technological Educational Institute of Central Macedonia, Terma Magnisias, GR-62124 Serres, Greece

**Keywords:** Lecture improvements, Teaching approaches, Engineering education, Computer networking course

## Abstract

The recent economic crisis has forced many universities to cut down expenses by packing students into large lecture groups. The problem with large auditoria is that they discourage dialogue between students and faculty and they burden participation. Adding to this, students in computer science courses usually find the field to be full of theoretical and technical concepts. Lack of understanding leads them to lose interest and / or motivation. Classroom experience shows that the lecturer could employ alternative teaching methods, especially for early-year undergraduate students, in order to grasp their interest and introduce basic concepts. This paper describes some of the approaches that may be used to keep students interested and make them feel comfortable as they comprehend basic concepts in computer networks. The lecturing procedure was enriched with games, magic tricks and dramatic representations. This approach was used experimentally for two semesters and the results were more than encouraging.

## Introduction

Lecture is the traditional method of teaching. Through a lecture the teacher is capable of providing the maximum flow of information to students in a controlled and well structured environment. This procedure is well fitted for large auditoria where personal interaction and hands-on approach are limited. A lecture allows the instructor to precisely determine the aims, content, organization, pace and direction of a presentation, expose students to unpublished material, and can complement and clarify text material. Moreover, with well planned lectures teachers can envisage and plan for almost anything that may happen in a classroom.

However, lectures are generally prepared from the instructor’s point of view, and the student’s need for interaction with the instructor is not addressed. In fact, lack of interaction is considered as one of the major limitations of the traditional lecture (Munson 1992). McIntosh (1996) observes that lecturing is frequently a one-way process unaccompanied by discussion, questioning or immediate practice, which makes it a poor teaching method. Due to this one-way communication the lecturer must make a conscious effort to become aware of each student’s problems or their ability to understand the content without verbal feedback. Moreover, experience shows that when students have copies of the lecture notes or a textbook, a significant percentage would prefer reading them rather than attending classes that offer little or no interaction.

So why do we lecture? McIntosh posed this interesting question in 1996. Most educators learn how to teach based on their experiences as students. Traditionalism perpetuates the lecture as a passive, one-way method of transferring information. The lack of faculty training in presenting effective lectures, rather than the method itself, may be the greatest weakness of the lecture (McIntosh 1996).

Another important issue is the diversity and complexity of student learning styles and processes. Students’ learning styles vary, and a teacher may confront a complex mixture of students in a single class. Felder and Silverman (1988) described some of the varied learning preferences of students. He classifies students as “visual learners”, who prefer to study graphs, models and pictures, “verbal learners” who seem to learn best from written materials, “tactile” and “kinaesthetic” learners that prefer real-life connections to the topic rather than theoretical approaches, and “sensing learners” who are tactile learners who favour subjects that allow them to work with their hands. Another division is made on the basis of how students comprehend a subject. So, there are “active learners” who tend to retain and understand information best by discussing or applying it or explaining it to others and “reflective learners” who prefer to think about it quietly first. There are, also, “global learners” who are excellent in synthesis and seem more likely than others to see a project as a whole, while on the other hand “sequential learners” are good with the analysis of concepts because they learn linearly. Since most classes are organized sequentially, this last kind of learner excels in the typical college class (Felder and Silverman 1988; Felder and Soloman). The average college teacher is much more likely to be sequential, verbal and reflective than his or her students are. To our knowledge there is at least one active research community in Europe that attempts to advance understanding on the theory and application of cognitive and learning styles in higher education as well as other contexts. This is the European Learning Styles Information Network (ELSIN) and a presentation of its work may be found in a recent paper by Evans et al. (2010).

The role of cognitive and emotional unconscious processing in mentally integrating visual and verbal instructional materials is dealt with in a recent review on human learning processes (Kuldas et al. 2013). It is concluded that conscious mental integration does not happen all the time, nor does it necessarily result in optimal learning. Students regardless of age and/or level of experience cannot always have conscious awareness, control, and the intention to learn or promptly and continually organize perceptual, cognitive, and emotional processes of learning. The review suggests that the understanding of unconscious learning processes and how mental associations are activated would assist in presenting students with spatially-integrated verbal and pictorial instructional materials as a way of facilitating mental integration and improving teaching and learning performance (Kuldas et al. 2013).

To enhance the complexity of teaching for the contemporary lecturer one should also take into account the cultural diversity in modern higher education environments. Increasing cultural diversity in the student body has become characteristic of tertiary and business higher education. The number of foreign tertiary education students enrolled outside of their country of origin more than doubled during the 2000–11 period, with an average annual growth rate of almost 7%. Over the past three decades, the number of students enrolled outside their country of citizenship has risen dramatically, from 0.8 million worldwide in 1975 to 4.3 million in 2011, a more than fivefold increase (OECD 2013). Fostering cultural inclusiveness and learning in culturally mixed classes is yet another complex task for the teacher and faculty needs continual professional development, and institutional support in intercultural competence development (Mak et al. 2014).

In order to cope with such diverse audience and to grasp students’ interest and enhance comprehension teachers usually embellish their lectures with examples and metaphors. Nevertheless, a student’s attention easily disappears after a few minutes of lecture.

An alternative approach is to make the teaching procedure more interactive by encouraging student participation. Although the quantity of information that reaches a student through participatory teaching methods is less, the quality of learning and the comprehension are significantly enhanced (Rossetti and Nembhard 1998). Student-centered methods, e.g., discussions or laboratories, require the instructor to deal with unanticipated student ideas, questions and comments. Thus, the instructor is able to influence students when they are actively working with the material.

Although student-active methods have been recently emphasized, their essence goes back to ancient Greece and the Socratic Method. Classical Greek philosopher Socrates used this inquiry and debate method with his students, opposing their point of view, in order to stimulate critical thinking and strengthen one’s ideas or lead him to an illuminated discovery of new ideas.

Today, participatory teaching uses methods like debates, oppositional discussion, games, role playing, presentations, small group discussions, brainstorming, collaborative learning, etc.

However, the majority of modern bibliography refers to the application of such methods in secondary education, or the use of electronic games in all levels of education. A literature review, up to 2004, on games and learning introduces to current thinking about the role of computer games in supporting children’s learning inside and out of school (Kirriemuir and McFarlane 2004). The use of electronic/video games in higher education is either reported as learning aiding environments for other disciplines or as projects for computer science students in order to motivate them in advanced programming courses (Bayliss 2009; Kurkovsky 2009). Their use is also expected to directly provide massive (and massively effective) parallel education in science and engineering (Mayo 2007). Little has been written, however, on how participatory teaching methods may be applied on other advanced computer science topics, such as computer networks, either as case studies or from a didactics perspective (McGuffee 2004; Shifroni and Ginat 1997).

The present paper attempts to provide several examples on how games, metaphors, and even “magic” can be used in a computer networks course in order to present to students the essence of technical topics, keep them interested or even make them have fun during a technical lecture. Classroom experience shows that these approaches, if used appropriately, enhance students’ involvement, interest and comfort. These unorthodox techniques can keep students attentive and target preferred learning styles (Stamm 2008). Moreover, they may also be applied not only in introductory higher education courses but in relevant secondary education ones. Adding to this, computer networks have became an essential part in computer science related curricula, driven by the great impact of internet, mobile and wireless technologies in everyday life.

The paper proceeds as follows. In the second Section relative paradigms in modern literature are presented. In Section 3 personal classroom experience and examples of how such alternative approaches are used in a computer networks course are given. In the fourth section a discussion of thoughts on issues that arise is made and in Section 5 conclusions are drawn.

### Previous and related work

Developing an interesting learning environment requires substantial creativity by the instructor. Many unorthodox teaching methods have been developed. If one places all these methods on a continuum then on the one end the traditional lecture is placed while on the other end one may find active student group work such as theatrical performances on how machines or protocols work. Some of the attempts to apply such methods in computer networks teaching are described below.

#### Drama and games in computer science classroom

One goal of participatory teaching methods is to engage students as active learners. McGuffee (2004) reports how he uses student led drama to teach three different reliable data transfer protocols in his computer networks course. These are namely the Alternating Bit, the Go-back-N and the Selective Repeat protocols. Traditional aids, when teaching networking protocols, are graphs, finite state machines and simulations. Classroom experience shows that confronting an average student with a finite state machine diagram may end up a tragedy! So, in order to give his students the opportunity to actively explore the challenges involved in reliable data transfer, McGuffy had them present the three different protocols in theatrical format. His novelty is that he leaves his students completely free to devise a way to dramatically present their assigned protocol.

Shifroni and Ginat (1997) also describe a game they use to teach communication protocols. The task is to understand a basic protocol of the Data-Link layer, namely the Stop-and-Wait protocol. In their case, the game is more formally defined and uncertainty is simulated by throwing a dice. The students act as the protocol components and are partitioned into five groups. The sending and receiving groups are placed in separate rooms; senders and receivers communicate by transmitting special paper forms via the physical layer group. The enactment used by students in this activity reinforces the computer science concepts and aids the learning process due to its unorthodoxy.

Hamey (2003) proposed the Security Protocol Game as a highly visual and interactive game for teaching secure data communication protocols. Students may use the game to simulate protocols such as the Transport Layer Security and the Pretty Good Privacy and explore possible attacks against them. Through the game the key issues of confidentiality, integrity, authentication and non-repudiation in secure data communications are revealed. The game is suitable for use in tertiary as well as professional education courses for managers and information technology students at all levels. This approach is the most controlled compared to the previous two. The instructor may even provide a playing kit, with appropriately coloured envelopes, rules, instructions, etc. Due to its nature it may only be performed by small student groups.

#### Analogy, exaggeration and extended analogy

Although student involvement is wanted, instructors must lecture. So, it is vital for them to use creative ways of teaching to keep the class interested and aid students’ understanding. Matocha et al. (1998) start from the familiar use of analogy and exaggeration, and propose the concept of extended analogy as a combination of the two. When an instructor creates an extended analogy for a topic, the analogy helps students by mapping the topic to familiar understandings, while the exaggeration enhances the ability to recall it. So, the analogy becomes an easy to remember story rather than a technical concept. The presentation of an extended analogy requires a certain amount of drama and improvisation skill. These creative approaches to teaching reinforce concepts, allow for easy recall of ideas, and capture students’ attention.

#### Properties of appealing teaching games

An equally interesting and important subject is the special properties an appealing teaching game should have. Malone (1980) discusses what makes things fun to learn. Although he describes his intuitions about what makes computer games fun, his ideas may also be applied on games used in the classroom. According to him the three essential properties are: challenge, fantasy and curiosity. Challenge is connected to the game’s obvious goal whose attainment is however uncertain. Achievement of a goal makes people feel better about them and enhances one’s self-esteem. Challenge refers to what a player, i.e. a student, can do. Fantasy is what makes things fun. It is actually the mapping of the player’s progress to some fantastic goal. Curiosity is mostly connected to the motivation to learn, and is independent of any goal-seeking or fantasy fulfilment. The learner’s curiosity will be stimulated only if the game has an optimal level of informational complexity. That is, the game environment or task should be neither too simple nor too complicated. Games should be novel and surprising but not completely incomprehensible.

### Our experience with the application of alternative teaching approaches

This work reflects the experience earned from teaching an introductory computer networks course to second year students in a higher education institute. These students study in an Informatics and Communications Department and begin with little or none theoretical background on the subject.

The task is to investigate whether the enrichment of the lectures with lively analogies and games can attract students’ interest and boost the learning procedure. Three examples follow that exhibit how games, magic and drama have been embodied in the lectures towards these tasks.

It should be noted, that due to the Greek Education System and Lyceum (Greek equivalent to High School) curricula, students that are admitted to Sciences and/or Engineering University Departments come with a good background on Mathematics (Algebra, Euclidean Geometry, Trigonometry, some Calculus, and basic Statistics), Physics, and Chemistry. They also have a limited background on Computer Science which is mostly on familiar software applications and algorithmic thinking. Any prior knowledge on more technical issues like Computer Networks can only be attributed to self study due to personal motivation and interest. Nonetheless, their level on Mathematics can be considered sufficient for an introductory Computer Networks course. Some required knowledge on Probabilities and Information Theory is usually provided by the University curricula in the first or early second year (third semester) of their studies.

#### Games

**Figure 1:**
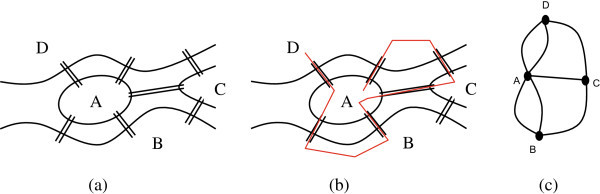
**The problem of the bridges of Konigsberg. (a)** A drawing of Konigsberg **(b)** An incomplete stroll around Konigsberg **(c)** A graph equivalent.

There are many simple games that can be played even on a whiteboard and need no special playing kits or specialized computer software. An example is all those games that one can play when he teaches graph theory. Graph theory is the study of graphs which are mathematical structures used to model pairwise relations between objects like the components of a communications network.An introductory graph theory course often starts with historic reference to Leonard Euler’s visit to the city of Konigsberg, which is situated on the Pregel River. Today, the city is named Kaliningrad, and is a major industrial and commercial centre of western Russia. The river Pregel flowed through the town, creating an island, as in the following picture (Figure 1a). Seven bridges spanned the various branches of the river, as shown.

**Figure 2:**
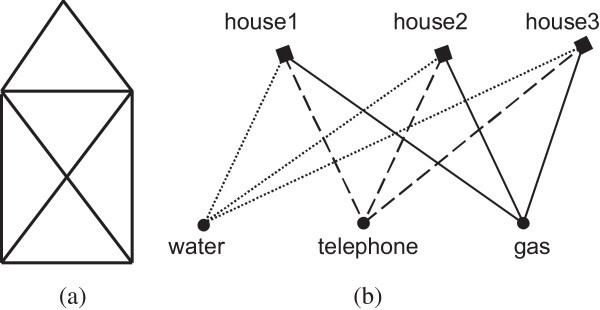
**Two well known graph related problems. (a)** The little house and **(b)** The three utilities problem.

It is said that the trendy talk of the time (18^th^ century) was whether it was possible to go for a stroll through the city in such a way that one crosses all bridges one and only one time. Figure 1b shows a wrong attempt. Euler observed that the size of the bridges or the land were irrelevant to the problem and altered the representation of the problem into that of Figure 1c, regarding the spots of land as points (vertices or nodes), and the bridges as paths (edges) between those points. This simplified version of the map of Konigsberg has the mathematical essentials of what is today referred to as a graph.The lecture starts by drawing the map of Konigsberg (Figure 1a), state the problem and then ask the students to try to find a solution. It is interesting to see how involved they become and how they interact as they try out possible solutions. Their first approach is always wrong as they underestimate the problem, especially those who try it by heart. If the teacher nicely challenges their abilities they get even more involved and after a couple of misses they stop playing aimlessly and attack the problem mentally. It is the time that some of them observe that if a bridge was missing or a new one could be build then the problem has an obvious solution. This is a critical step towards the abstraction of the problem. Identifying the properties of that bridge leads to the notion of the order of a vertex and hopefully to the reasoning of why the specific problem can not be solved.It is interesting that Euler never published an algorithm for finding the solution, but only provided a method of determining if one existed or not. Euler's solution to the problem involved the observation that when a vertex is visited in the middle of the process of tracing a graph, there must be an edge coming into the vertex and another edge leaving it; and so the order of the vertex (i.e. the number of edges ending or originating from the node) must be an even number. This must be true for all but at most two of the vertices, the one you start at, and the one that you end the walk. So, a connected graph is traversable if and only if it has at most two vertices of odd order. Now, a quick look at the graph in Figure 1c shows that there are more than two vertices of odd order, and so the desired stroll around Königsberg is impossible or in graph theory’s jargon the graph does not have an Euler circuit. Some of the students reason their conclusions in exactly the same way that Euler did. When they are told that one of the most pioneering mathematicians in human history gave the same explanation to the problem with them, their self esteem goes really high. However, establishing graph theory was only a fragment in the whole Euler work.As soon as students familiarize with the notions of graph theory it is easy to understand why they can draw the little house in Figure 2a, a game they are probably familiar with since elementary school days, without lifting the pencil from the paper.The next step is to introduce properties of planar graphs by playing similar games, e.g. to show why it is impossible to provide connection with three public utilities (e.g. water, electric power and telephone) for three adjacent houses without intersecting the service networks (Figure 2b). It is tempting to reference here a student’s comment who stated that he preferred a broadband internet connection to his home than water!

Several problems and games related to graph theory with their formal solutions may be found (Chartrand 1985). Games become easy or hard as the graphs change. Articulating this is an essential part of communicating the problem solving process. The relationships between the parts of a graph are an excellent basis for seeking patterns and relationships and for spatial reasoning. Moreover, when games are associated with stories, the stories show how an abstract mathematical object can be related to an everyday life situation (Casey and Fellows 1993).

#### Magic

Magic may be used for teaching the implications behind the Hamming Code. Hamming code is a binary linear error-correcting code named after its inventor, Richard Hamming (1915 – 1998). Hamming code can detect up to two simultaneous bit errors, and correct single-bit errors. Due to its simplicity it is a good example of error correcting code in telecommunications and is also widely used in computer memories (RAM) (Moon 2005).

**Figure 3:**
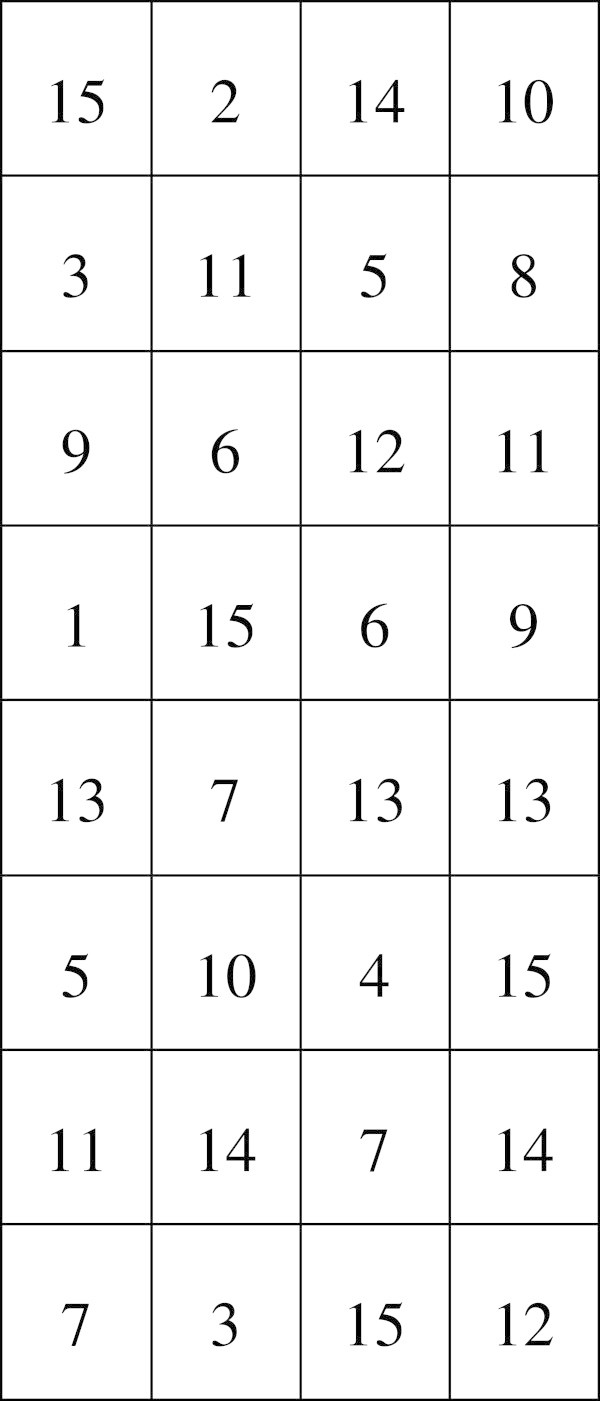
**A magic table.**

A didactic problem is how to reason the Hamming bit position and how to make students remember the positions and the procedure of estimating their values. When the teacher gets to this point then magic and exaggeration come into the scene. The teacher becomes a mentalist who has the hereditary gift of reading other peoples’ mind. In order to prove his abilities one may draw a magic table on the whiteboard, like the one depicted in Figure 3. Then the instructor may randomly ask students to think one of the numbers in the table and tell him the number of all the columns the number appears in. For example, a student may tell that the number appears in the first and third column, and then the teacher stares deep into the students’ eyes to read his or her mind and guess that he or she has picked number 5. After a couple of successful guesses the students try to find the trick. They first think that their instructor succeeded because he looked at the table, but after a couple more guesses with his back turned to the whiteboard, they suppose that he has memorized the position of the numbers. If the tutor interchanges a couple of numbers in the table, the class is becoming intrigued by his secret talent. This is when they stop playing and start thinking. Few notice the trick. Numbers are placed in specific columns. The 1 (2°) is placed somewhere in the first column, 2 (2^1^) in the second, 4 (2^2^) in the third and 8 (2^3^) in the fourth. All other numbers are in the columns that participate in a sum made up from the powers of number 2, which gives each number, e.g. 5 = 2° + 2^2^ appears in the first and third column, 11 = 2° + 2^1^ + 2^3^ appears in the first, second and forth column, and so on. Each column actually represents a place-value of the binary system.

This is a typical example of exaggeration which may also be used to introduce number systems, especially the one with base 2. The notion of magic is very appealing to students. Also, due to the coincidence of a contemporary TV series (i.e. The Mentalist) and a recent TV show (i.e. The Successor of Uri Geller) students find the mentalist concept very trendy and familiar.

#### Drama

The dominant data communications protocol in modern local area networks (LANs) is Ethernet. Trying to show the analogy between the Ethernet protocol (IEEE 802.3) and a polite conversation by means of a theatrical performance is really a task that not only teaches the nuances of Ethernet but also the essence of politeness.

Ethernet’s function is based on the CSMA/CD (carrier sense multiple access with collision detection) protocol which ensures that only one station is transmitting at any time over the common shared medium. If any transmitting station senses that another station’s signal is also present on the medium then it stops transmission, sends a jamming signal on the line to ensure that all other stations become aware of the signal collision, and then invokes a binary exponential back-off algorithm, i.e. it waits for a random number of time slots before attempting to retransmit (Tanenbaum 2002).

A direct analogy to Ethernet is an open and polite human conversation. Imagine a group of people sitting around a meeting table, where each one has equal rights to speak and there is no chairman. Whenever one wants to speak he is free to do so as soon as thoughts come to his mind. Of course, there is always a chance that another person is also ready to talk. If two people start speaking together then both should politely yield and wait a little before start speaking again.

Although it is easy to imagine a situation like that, it is difficult to theatrically present it (at least with inexperienced actors like students are). Without guidance it is difficult to activate concurrent talking and also to simulate the “wait a little” process.

Students sit one beside the other, all facing the whiteboard. Each student performs the part of a station and is assigned a number which represents its physical address. A slide presentation is projected on the whiteboard so anyone can see and synchronize. Whenever a slide changes then a “random” number appears which corresponds to the number of a student (i.e. the physical address of a station). Then this student starts reading loudly a sentence written on the slide, just under the number. This text line is the transmitted message. Some slides may have two different numbers. Then, both stations (students) with the corresponding addresses must simultaneously read the line under the numbers, and experience a collision. Now it’s time for the two stations to activate the back-off algorithm. That is, they each toss a die and read the outcome. Then they count a number of seconds equal to the outcome of their die and the one with the smaller number speaks first.

Students like to make corrections during the play. In fact, this is the desirable outcome of the procedure. The most common correction is on the approach that is used to simulate collisions, which is performed in a way that is not the right one. In real Ethernet networks stations back-off as soon as they detect a collision. In our approach the students read the entire text line. After a couple of examples some students grasp the difference, they correct the director (i.e. the instructor) and embody the correct approach in their performance. Another basic difference is that the binary exponential back-off algorithm is, as the name implies, binary. That is, when successive collisions happen then the possible outcomes of the die are doubled. In other words, after the first collision the random outcome is either 0 or 1, after the second collision the outcome is 0, 1, 2 or 3, and so on. When the students grasp this difference then it is the teacher’s turn to decide it is time to stop playing and continue lecturing. The basic nuances of the protocol have just been learned.

**Figure 4:**
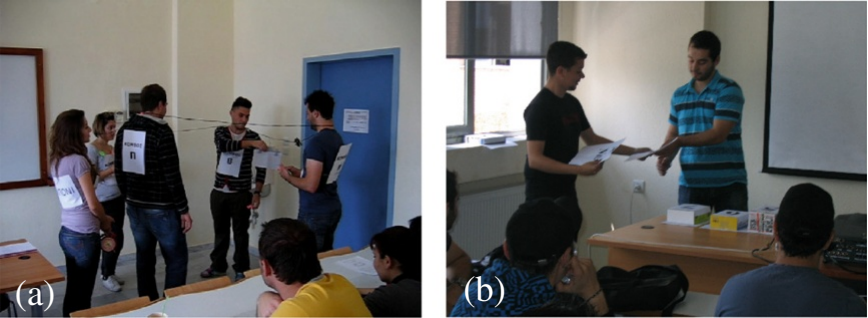
**Shots taken during the dramatic presentations of computer network protocols. (a)** Token Ring: messages are transmitted over the ring (wire) while the token (student on the left) holds an alarm-clock to keep the token holding time, **(b)** Acknowledgements arrive at transmitter’s site and packets (numbered boxes on the desk) wait for their turn during a presentation of the Go-Back-N protocol.

Students find the method very amusing and welcome the alternative teaching approach. Last semester a team of students offered to theatrically perform another network protocol that is usually being taught in conjunction with Ethernet, namely the Token Ring protocol. This is a much more controlled networking protocol which also has some ticklish points. Their performance presented the basic protocol characteristics but failed to point-out the details. However, the class was much more receptive to a lecture on the protocol details after the performance (Figure 4a). Another student group offered to theatrically present the Go-Back-N protocol (Figure 4b). For a detailed presentation of the two networking protocols the reader is referred to (Tanenbaum 2002). Students were so excited and thrilled by this deviation from traditional lecture that the news spread out quickly and many of the fellow teachers were also intrigued by the students’ response.

### Method of evaluation of the new lecturing approach

The lecturing procedure, on what is otherwise a theoretical or technical subject, was enriched with games, magic tricks and dramatic representations. This approach was used experimentally for two semesters and the results were more than encouraging. It should be noted that the course is also supported with laboratory exercises as well as an e-learning environment where the lecture slides are provided along with video recordings of the lectures and an open forum for discussions. The main difference with past years was that the lectures were systematically enhanced with the aforementioned practices.

Regarding the passing rate of the course there was only minor improvement compared to the previous two semesters. The success rate rose from 45% to 47.2% which may be contributed to a random fluctuation. But the real gain was in student enthusiasm and involvement. This was apparent throughout the semester and was also backed by the students of the following semester who started the course with one question: shall we also play theatre?

Students’ positive reaction was also made apparent during the annual teaching quality assessment of the Department. This is performed annually by means of the course evaluation questionnaire presented in Table 1. The questionnaire is designed to provide faculty members with useful feedback regarding ways to enhance student learning in a course. It consists of 23 questions divided into five categories; namely the evaluation of course materials (resources, assignments, and assessments), evaluation of the course, evaluation of the teaching, evaluation of laboratory teaching, evaluation of student involvement/engagement. The response scale runs from 1 to 5, with 5 being the highest (strongly agree), i.e. (1) Strongly Disagree, (2) Disagree, (3) Neutral, (4) Agree, (5) Strongly Agree.

**Table 1:** **Course evaluation questionnaire**

*Question No.*	*Question*
	*Evaluation of course materials (resources, assignments, assessments)*
1	The course material taught during the semester was well organized
2	The additional material used (photocopies, videos, slides, etc.) helped towards a better understanding of the subject matter
3	The books (if any) were distributed in time
4	The books (and any additional material) were helpful.
5	There was easily accessible and relevant research material in the library
	*Evaluation of the course*
6	The course was difficult as regards the semester it is being taught
7	The aims of the course were attained
8	In cases where there were written and/or oral assignment/s: There was guidance from the instructor
9	In cases where there were written and/or oral assignment/s: You were given a chance for improvement
10	In cases where there were written and/or oral assignment/s: This assignment helped you to understand the particular subject matter
	*Evaluation of the teaching*
11	The instructor succeeded in stimulating students' interest for the course
12	The instructor uses effective teaching methods and examples that enhance my learning.
13	The instructor encouraged students to join in discussions in a way that helped them to participate and develop knowledge/capabilities
14	The instructor was consistent in keeping class and office hours, provided timely feedback on projects
15	The instructor was receptive/open to students’ questions
16	In general, the overall performance of the instructor was very good
	*Evaluation of laboratory teaching*
17	In case there were laboratory assignments/exercises: the difficulty level relevant to the semester that the course is being taught
18	In case there were laboratory assignments/exercises: the laboratory textbook and other teaching material were helpful.
19	In case there were laboratory assignments/exercises: the guidance provided during the laboratory hours was helpful and sufficient
20	In case there were laboratory assignments/exercises: the laboratory equipment was sufficient
	*Evaluation of student involvement/engagement*
21	I attend class regularly.
22	I always participated in class discussions, projects, written and/or oral assignments
23	On average, I have spent ________ hours per week doing work outside of class for this course. (0–1 hour (1), 2–4 hours (2), 5–6 hours (3), 7–8 hours (4), 9+ hours (5))

The students answers to the questionnaire were evaluated for three semesters. The first semester was the one prior to the application of the new approaches to the teaching procedure (pre-intervention). 34 undergraduate students (Second Year) who enrolled the course filled out the questionnaires. The second semester was the one where the new teaching approach was first introduced (1st intervention semester). 33 undergraduate students filled the questionnaires. In the third semester (2nd intervention semester), 43 students filled out the questionnaires and it was used to verify the change.

One of the things that are being explored by means of the questionnaires is the teacher’s ability to use effective teaching methods and examples that communicate ideas and knowledge to the students (Table 1: Question 12). The median of the answers to the specific question rose from 4.36 to 4.62 in a scale from 1 to 5. The corresponding median for the whole Department was 4.1 (Table 2). Another question explores whether student involvement is encouraged during the lectures (Table 1: Question 13). There was also a remarkable raise in the median which rose from 3.68 to 4.32 in the same scale. The median for all the courses in the department was 4.05 (Table 2). Last, as regards the success to stimulate students' interest for the course (Table 1: Question 11), the corresponding figures were 3.62 (prior to intervention), 4.06 (after intervention), and 3.56 (for the Department) as is shown in Table 2. In the same Table (Table 2) the figures for the second intervention semester are also given.

**Table 2:** **Average response to teaching quality and accomplishment related questions for the intervention and the pre-intervention semesters**

*Question*	*Course's average*	*Course's average*	*Course's average*	*Department's average score*
	*(previous semester)*	*(1st semester of intervention)*	*(2nd semester of intervention)*	*(1st semester of intervention)*
The aims of the course were attained.	3.6	3.75	4	3.50
The instructor succeeded in stimulating students' interest for the course.	3.62	4.06	4.2	3.56
The instructor uses effective teaching methods and examples that enhance my learning.	4.36	4.62	4.35	4.10
S/he encouraged students to join in discussions in a way that helped them to participate and develop knowledge/capabilities	3.68	4.32	4.5	4.05
In general. the overall performance of the instructor was very good	3.92	4.16	4.45	3.89

**Figure 5:**
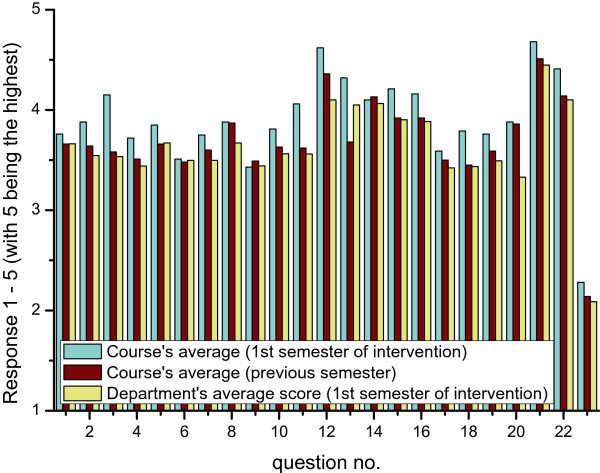
**Response scores to the course’s questionnaires before (second bar) and after (first bar) the intervention.** The Department’s average score for the first semester of intervention is also depicted (third bar).

As a whole the average evaluation of the course, for all the 23 questions in the questionnaire, also rose during the two intervention semester compared to the course’s evaluation prior to the intervention, as well as the average evaluation for all the courses in the Department. Figure 5 depicts this comparison. Student’s paired *t*-tests (*alpha* = 0.01) were performed to compare the mean course evaluation before and after the intervention. The null hypothesis was that there was no change in the mean. For the semester before the intervention the evaluation score for the course had *M* = 3.69 and *SD* = 0.44. For the semester where the new teaching approaches were applied the course’s score had *M* = 3.90 and *SD* = 0.48. This difference was statistically significant, *t*(22) = 5.54, *p* = 1.43E-05. In other words there was a significant improvement in the course’s evaluation by the students.

As regards the comparison of the course’s score against the mean score of all the courses offered by the Department, the results also showed significant difference. In particular, the course’s score for the semester where the new teaching approaches were applied, had *M* = 3.90 and *SD* = 0.48. The corresponding figures for the Department, for the same semester, were *M* = 3.63 and *SD* = 0.44. This difference was statistically significant, *t*(22) = 7.90, *p* = 7.25E-08. In other words, the course was evaluated significantly higher than the mean evaluation of all the courses in the Department. In Table 3, the statistics *M* and *SD* for the three sequential semesters are given. Informed consent for publication was obtained.

**Table 3:** **Mean and standard deviation of the course’s evaluation one semester before and two semesters after the intervention**

	*Course's average*	*Course's average*	*Department's average score*	*Course's average*	*Department's average score*
	*(previous semester)*	*(1st semester of intervention)*	*(1st semester of intervention)*	*(2nd semester of intervention)*	*(2nd semester of intervention)*
Mean (M)	3.69	3.90	3.63	3.95	3.49
Standard Deviation (SD)	0.44	0.48	0.44	0.57	0.42
Number of questionnaires	34	33	2418	43	3144

## Discussion

Due to the recent economic crisis, many universities are compelled to create large lecture groups of students. Large auditoria burden the teaching process and the lecturer is typically confronted with the lack of student participation during his or her lectures. This absence of creative dialogue between students and faculty seems to emerge as a common characteristic in today’s higher education. This work presents some of the techniques that are employed to transform a traditional lecture to a more compelling student experience.

From a pedagogical point of view a teacher is expected to address all types of students regardless of their different learning styles and balance his teaching approach accordingly. However, the average college teacher is much more likely to be sequential, verbal and reflective than her students are (Felder and Silverman 1988). So, she tends to approach topics in a sequential manner and assist her teaching with appropriate textbooks.

Nevertheless, an instructor should try to motivate learning regardless from ones own tendencies. It is important for students to confront with knowledge “that makes sense”. Those in technical professions, like computer science and engineering, want to know how related is the theoretical knowledge they get with the skills an employer will expect from them. The material that is being presented should be related to what has already been taught, related to material in other courses and, if possible, connected to the student’s personal experience. Material that emphasizes practical problem-solving should be balanced with material that emphasizes fundamental understanding, and sensing information like facts, real world data, exercises, experiments, should be balanced with abstract concepts like theories and models.

Lectures should be enriched with pictures, schematics, graphs and simple sketches. When possible it is nice to show films (nowadays the Internet is full of short technical films from the industry and manufacturers), and provide demonstrations and /or hands-on approaches (e.g. call a practitioner whose profession relates to the course in order to give a short lecture, exhibit how a specialized instrument works or consult about particular skills needed in his profession).

Group projects for homework or class give students the option of cooperating to the greatest possible extent. Active learners generally learn best when they interact with others; if they are denied the opportunity to do so, they are deprived of their most effective learning tool.

Let students do something different besides transcribing notes from the whiteboard. Ask questions, give relevant exercises, and let them do some brainstorming in groups. Instructors can, also, help students become active learners by motivating them with open-ended questions, puzzles, and paradoxes (Felder and Silverman 1988).

Some common sense guidelines for making lectures more interesting and vivid are presented in (Bayliss 2009). McGuffee (2004) also gives several tips for implementing dramatic exercises in classroom. Garris et al. (2002) discuss several aspects of incorporating games in teaching, present an input-process-output model of instructional games and discuss the implications of this approach for the design and implementation of effective instructional games. Key elements for successive incorporation of such methods in classroom are a conducive school environment and the instructor’s level of experience.

The teaching experience dictates that magic and games are easier to be embodied in a traditional lecture than drama. Their use is mostly a matter of the teacher’s personality, creativity and ability to be less strict when lecturing. Magic and games may directly be incorporated into the lecture and do not spend significant amount of the course’s time burdening other topics that need to be addressed later. It should be stated that such procedures may have already been used from some teachers unwittingly and is interesting to observe yourself in order to identify and enhance this ability.

On the other hand, enhancing the teaching procedure with drama is a game for two. It depends on the students’ will for active involvement and is a bigger project for the teacher. A dramatic representation of a subject needs to be prepared, orchestrated and directed. It also needs time to be presented by the students which significantly reduces the time left to cover other topics. However, we believe that the gain in the learning procedure is higher because students are actively involved and need to comprehend the subject before transforming it into a theatrical play.

Another difficulty with the dramatic representation of a technical subject is that it is hard to be applied in large auditoria. However, such approaches may prove very helpful in small classrooms and we believe that are very appropriate for introductory computer network courses in secondary education.

If motivating students is of the most important things a teacher should do, rewarding them and applauding their efforts is the other most important action. Sometimes a reward is the most powerful motivation.

Most important, professors should understand the importance of encouraging students to ask and answer questions, not only inside but also outside the class. Nowadays, Web 2.0 enhances collaboration and interaction, and modern communication media, e.g. e-mails, chat rooms, forums, social media as well as asynchronous e-learning environments, offer a plethora of means to communicate, participate and collaborate. We are also witnessing a global tendency to open courses delivery. Massive open online courses (MOOCs) have appeared, like Coursera, Udacity and edX (Kolowich 2012). The success and acceptance of such efforts open a whole new perspective in education. These approaches liberate people from time or space barriers when they also provide access to the physically impaired. Adding to this, multimedia and flash presentations of a topic are able to make it vivid and easier to comprehend.

One should be aware though, that an online learning environment adds new barriers to teacher-student interaction. One of the tools that the lecture has in order to interpret comprehension level of students is the facial expression of the students. A real classroom enables live face-to-face communication (Sathik & Sofia 2011); many virtual classrooms aim to implement this by having regularly scheduled chat room with video conferencing interactions where students can interact with each other and the lecturer as they would in a real classroom. Facial expression is reported as the most frequently used nonverbal communication mode by the students in the virtual classroom and facial expressions of the students are significantly correlated to their emotions which helps to recognize their comprehension towards the lecture (Sathik and Sofia 2013). However, a teacher, especially one at higher education where students come from different cultural backgrounds, should be aware that perception of facial expressions differs across cultures and these differences could cause cross-cultural misunderstandings (Jack et al. 2012; Marsh et al. 2003).

In general, participation approaches should make students feel comfortable and not intimidated. All shall be given equal opportunities to consult with faculty, tutors and other teaching personnel.

Comfort level in a computer science class has been identified as the best predictor of success in a course (Wilson and Shrock 2001), and a teacher must utilize all means of alternative teaching to keep the student’s interest alive, and all means of participation to motivate dialogue in order to counter fight the effects of massive higher education. This is a new era we are entering in and it is a teacher’s duty to be always alert, communicable and open to fresh ideas.

## Conclusions

This paper describes some of the approaches that may be used to keep students interested and make them feel comfortable as they comprehend basic computer networking concepts. Towards this task the lecturing procedure was enriched with games, magic tricks and dramatic representations. This approach was used experimentally for two semesters and the results were more than encouraging. The experience gained dictates that magic and games are easier to be embodied in a traditional lecture than drama. On the other hand, a dramatic representation of a subject needs to be prepared, orchestrated and directed, and thus it is easier to be managed within smaller student groups. However, the gain is higher because students are actively involved. Such approaches may prove very helpful when one tries to get students involved and we believe that are very appropriate for introductory computer networks courses not only in higher but in secondary education as well.
